# A Truncated Galectin-3 Isolated from Skin Mucus of Atlantic Salmon *Salmo salar* Binds to and Modulates the Proteome of the Gram-Negative Bacteria *Moritella viscosa*

**DOI:** 10.3390/md18020102

**Published:** 2020-02-04

**Authors:** Deepti Manjari Patel, Yoichiro Kitani, Kjetil Korsnes, Martin Haugmo Iversen, Monica Fengsrud Brinchmann

**Affiliations:** 1 Faculty of Biosciences and Aquaculture, Nord University, 8049 Bodø, Norway; deepti.m.patel@nord.no (D.M.P.); yki@se.kanazawa-u.ac.jp (Y.K.); kjetil.korsnes@nord.no (K.K.); martin.h.iversen@nord.no (M.H.I.); 2 Noto Marine Laboratory, Division of Marine Environmental Studies, Institute of Nature and Environmental Technology, Kanazawa University, Noto-Cho, Ishikawa 927-0553, Japan

**Keywords:** galectin-3, bacteria, proteomics, Gram-negative, lectin, innate immunology, mucosal immunology, agglutination, hemagglutination, multidrug transporter

## Abstract

The mucus of fish skin plays a vital role in innate immune defense. Some mucus proteins have the potential to incapacitate pathogens and/or inhibit their passage through the skin. In this study the aim was to isolate and characterize galectin(s), β-galactosides binding proteins, present in skin mucus. A novel short form of galectin-3 was isolated from Atlantic salmon skin mucus by α-lactose agarose based affinity chromatography followed by Sephadex G-15 gel filtration. Mass spectrometric analysis showed that the isolated protein was the C-terminal half of galectin-3 (galectin-3C). Galectin-3C showed calcium independent and lactose inhabitable hemagglutination, and agglutinated the Gram-negative pathogenic bacteria *Moritella viscosa*. Galectin-3 mRNA was highly expressed in skin and gill, followed by muscle, hindgut, spleen, stomach, foregut, head kidney, and liver. *Moritella viscosa* incubated with galectin-3C had a modified proteome. Proteins with changed abundance included multidrug transporter and three ribosomal proteins L7/12, S2, and S13. Overall, this study shows the isolation and characterization of a novel galectin-3 short form involved in pathogen recognition and modulation, and hence in immune defense of Atlantic salmon.

## 1. Introduction

The fish skin is a living, mucosal tissue where the mucus is essential to protect the animal [1]. Fish skin mucus is a rich source of molecules with immune relevant functions [2]. Lectins are proteins that recognize sugar moieties through their lectin domain(s) and hence bind to carbohydrates, glycolipids and glycoproteins. Lectins can also bind directly to proteins [3] or adenine [4] in a non-carbohydrate dependent manner.

The diversity in binding partners results in multiple functions of lectins. In mucosal surfaces they can contribute to immune defense by agglutinating pathogens and inhibit uptake [5]. However, lectins can also contribute to pathogenesis probably by stimulating pathogen uptake [6]. Extracellular functions of lectins include opsonization, cellular uptake through endocytosis and phagocytosis, start of the lectin pathway of the complement system and cell-cell interaction. Intracellularly lectins have diverse roles including roles in protein folding [7], in organelle movement, in uptake (into endocytic, autophagic and lysosomal organelles) and subsequent destruction of virus and bacteria found in cytosol [8], as well as roles in mRNA splicing and stability [9]. Lectins were first purified and characterized from plants; later lectins have been reported from microorganisms to humans, including aquatic animals such as teleosts [10,11,12].

We have previously isolated and characterized β-galactoside binding galectin 1-1 and galectin 1-2 [13] from Atlantic cod *Gadus morhua* skin mucus. In the present study we aim to isolate galectin from Atlantic salmon skin mucus by affinity purification by lactose-binding. Three groups of galectins exist, the prototype galectins where the whole protein is essentially a globular carbohydrate binding domain (such as in galectin-1), chimera type galectins with a N-terminal tail in addition to the carbohydrate binding domain (galectin-3) and tandem repeat galectins where there are two carbohydrate binding domains (such as galectin-4). Previously characterized skin and/or skin mucus galectins AJL-1 from Japaneese eel (*Anguilla japonica*) [14], Congerin I and II from conger eel (*Conger myriaster*) [15], and galectin 1-1 and 1-2 from Atlantic cod (*Gadus morhua*) [13] were all prototype galectins, hence the isolation of prototype galectin(s) from Atlantic salmon skin mucus was expected to be the outcome of this study.

Further we aim to identify and characterize the isolated protein(s) and we hypothesize that galectin present in skin mucus can bind to and affect bacteria relevant for skin disease. The bacteria used *Moritella viscosa* (formerly named *Vibrio viscosa)* cause skin ulcers, winter ulcers, in Atlantic salmon at low water temperatures [16].

## 2. Results

### 2.1. Isolation of Lactose Binding Protein from Atlantic Salmon Skin Mucus

**Figure 1:**
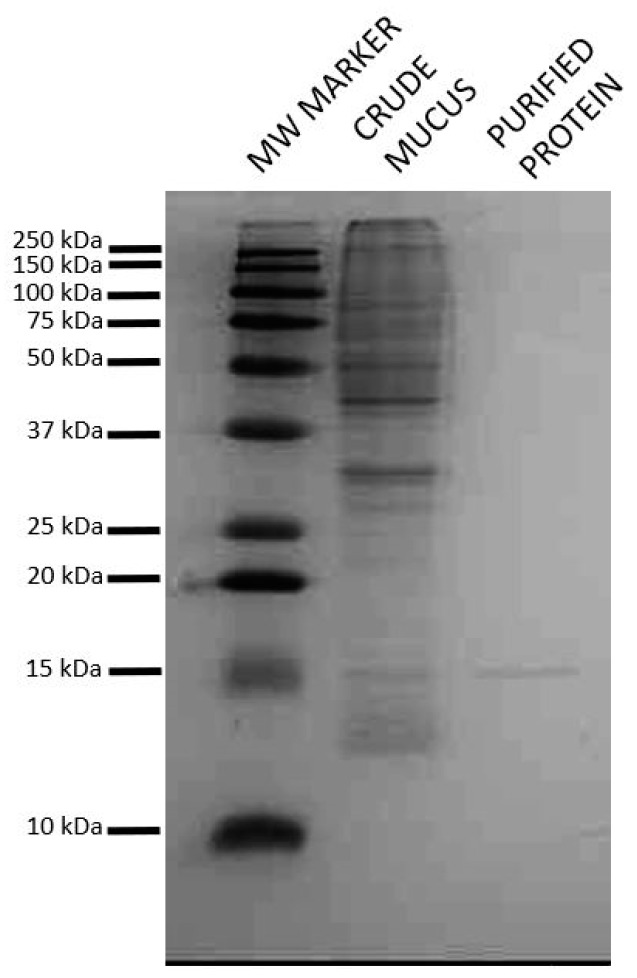
Confirmation of the purity of the protein isolated from Atlantic salmon skin mucus. Lactose binding protein from Atlantic salmon skin mucus was isolated by α-lactose agarose. The desalted eluate was run on a 15% SDS polyacrylamide gel under reducing conditions. The gel was stained by colloidal Coomassie G-250. Precision Plus, Kaleidoscope^TM^ (Protein^TM^ Standards, Bio-Rad) protein marker was used as a molecular weight marker. A single band was observed.

Galectins bind to β-galactosides and affinity purification with α-lactose agarose followed by Sephadex gel filtration was used to isolate putative galectin(s) from Atlantic salmon skin mucus. SDS-page of the isolated protein showed a single band at 15 kDa (Figure 1). This molecular weight is close to that of galectin 1-1/galectin 1-2 from Atlantic cod [13], and hence indicated that the isolated protein was a prototype galectin consisting of only the carbohydrate recognizing domain.

### 2.2. Identification of the Isolated Protein as a Short Form of Galectin-3

**Figure 2:**
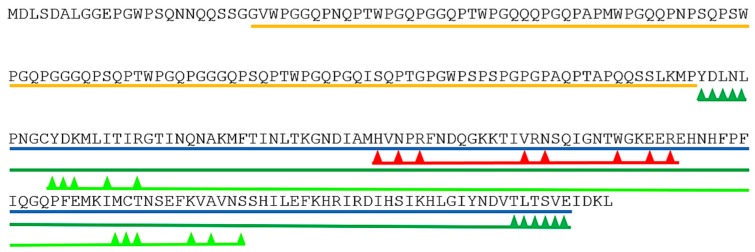
Conserved domains on [gi|213514684|ref|NP_001134305|] galectin-3. Information used in the figure is from www.ncbi.nlm.nih.gov/Structure/cdd/. Blue GLECT superfamily domain, red sugar binding pocket, green dimerization areas, light green putative dimerization domains, and orange PRK10263 domain. Arrowheads point to amino acids involved in sugar binding (red) or dimerization (green).

To identify the protein(s) present the band was excised and analyzed by mass spectrometry with ESI-Quad-TOF followed by Mascot search (http://www.matrixscience.com/). The band was shown to consist of one protein, identified as chimera type *Salmo salar* galectin-3 ([gi|213514684|ref|NP_001134305|]), with a mascot score of 220 and a peptide protein coverage of 19%. The unique peptides identified are found in Supplementary File 1. The galectin-3 protein in the database is 271 amino acids long with a molecular weight of 29,580 Dalton, twice the ~15 kD of the isolated protein. Two conserved domains in galectin-3 were found by searching the Conserved Domain Database (CDD, https://www.ncbi.nlm.nih.gov/cdd/). The first, the galectin’s galactose-binding lectin domain binds β-galactosides, such as lactose, and maps to the C-terminal part of galectin-3. The second domain, PRK10263, DNA translocase FtsK is provisional and maps to the N-terminal part of the protein. Dimerization areas are also present (Figure 2).

**Figure 3:**
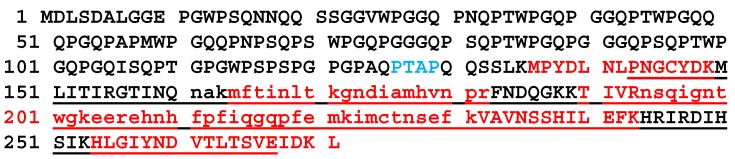
Peptides mapped to galectin-3 by mass spectrometry analysis of the Atlantic salmon skin mucus galectin-3. Underlined, the galectin domain. Amino acids highlighted in red were covered by Q-Exactive, the sequence in small letters was covered by ESI-Q-TOF. In blue the PTAP sequence.

The ESI-Quad-TOF identified peptides that were Mascot mapped to galectin-3 were all unique and in the C-terminal part of the protein from amino acid 161 to 231 (Figure 3). To increase the sequence covered by mass spectrometric analysis and to more precisely identify which part of galectin-3 was isolated from skin mucus, further mass spectrometric analysis was performed with Q-Exactive Quadrupole Orbitrap (Thermo scientific). The Q-Exactive protein coverage was 38.75% of the full-length protein with a Mascot score of 3739.77. Of the matched peptides 10 of 11 were unique and were all found from amino acid 136 to amino acid 271 of the full-length protein (Figure 3).

The part covered from amino acid 136 (methionine) to the C-terminal is 50.2% of the full-length protein (molecular weight 29.6 kD), this is in accordance with the protein weight of 15 kD observed in SDS-page (Figure 1) for the isolated lactose binding protein. The isolated protein is hence identified as the C-terminal part of galectin-3 (galectin-3C) with a theoretical molecular weight of ~15 kD, this part covers the galectin domain and the dimerization domains (Figure 2). In mammals it is shown that the sequence P(S/T)AP is needed for extracellular export of galectin-3 [17]. This sequence is present once in Atlantic salmon galectin-3 from amino acids 126 to 129 (Figure 3 in blue).

### 2.3. Tissue Distribution of the *leg3* Gene

**Figure 4:**
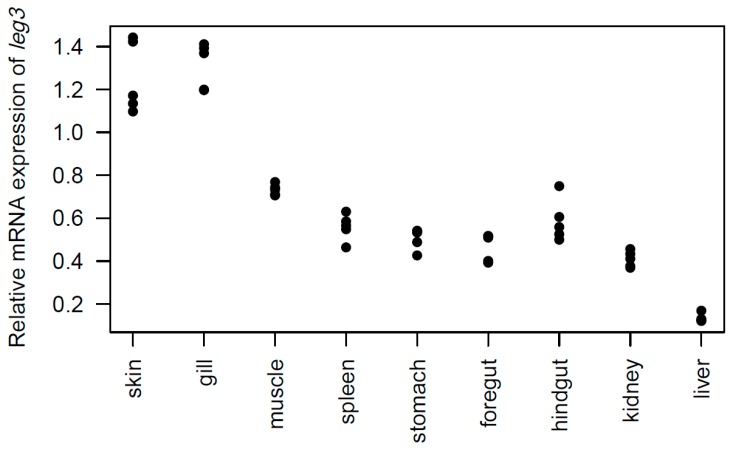
Tissue distribution of *leg3* in Atlantic salmon tissues. The plot shows mRNA expression of *leg3* relative to *EF1alfa* (*n* = 5). Values show the square-root transformed.

Primers were designed and reverse transcription qPCR was used to determine the tissue distribution of the *leg3* gene (coding for galectin-3) in skin, gill, muscle, spleen, liver, head kidney, stomach, foregut, and hindgut of Atlantic salmon. The expression of *leg3*, i.e., mRNA, was detected in all tissues tested (Figure 4). The expression was high in gill and skin, liver showed the lowest level of expression, and medium expression was found in the other tissues examined. The high expression of *leg3* in skin suggests local synthesis of skin mucus galectin-3C.

### 2.4. Hemaglutination Activity of Galectin-3C

**Figure 5:**
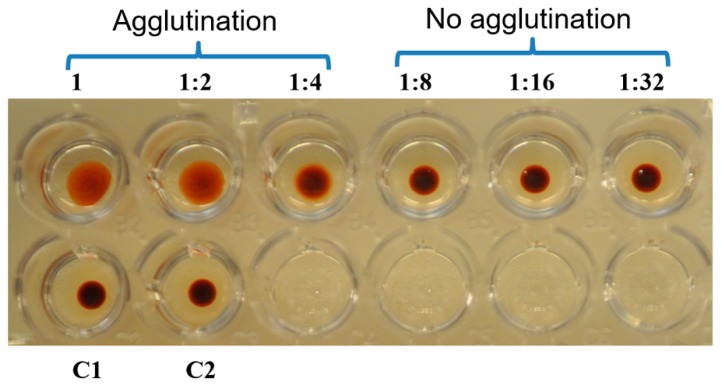
Hemagglutination and inhibition assay. The purified C-terminal half of galectin-3 (galectin-3C) (50 μg protein/ml final concentration) in upper left well was twofold serially diluted and mixed with horse blood (final well concentration 1%), BSA in PBS (final well concentration 0.25%), upper row. C1 is control with PBS instead of galectin-3C. C2 is control with 0.5 M lactose in the presence of 50 µg/mL galectin-3C), showing inhibition of hemagglutination.

Galectin-3C showed calcium independent (calcium was not added in the assay) hemagglutination activity at 50, 25, and 12.5 µg/ml and this activity was inhibited by 0.5 M lactose (Figure 5), confirming the lactose binding property of galectin-3C suggested by the lactose affinity column isolation.

### 2.5. Galectin-3C and Lactose Change the Growth Curves of Moritella viscosa 

**Figure 6:**
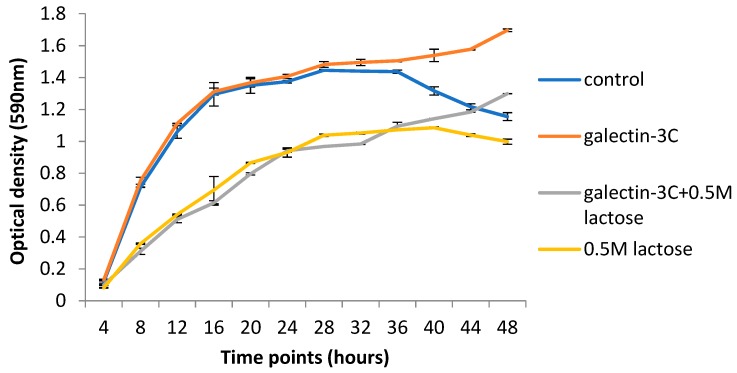
Growth curve of *Moritella viscosa* at 15 °C. *M viscosa* was grown in tubes under orbital shaking (200 rpm) at 15 °C in medium (3% TSB and 1.5% NaCl) with PBS (control) with or without 0.5 M lactose, or with galectin-3C (13 μg/mL) with or without 0.5 M lactose for the indicated time. Aliquots (*n* = 3) were removed at each time point and OD at 590 nm was measured. Error bars show SD. The figure is a representative figure from one out of 3 independent experiments with similar results.

Galectin-3C did not change the optical density of the *Moritella viscosa* culture in the log phase nor in the beginning of the stationary phase (Figure 6). Late in the stationary phase and in the death phase, however, the optical density was higher in galectin-3C containing samples compared to the PBS control samples. Incubation with lactose, both in the presence and absence of galectin-3C, led to lower optical density levels compared to the PBS control samples, suggesting growth inhibition in the presence of lactose. Late in the stationary phase galectin-3C with lactose increased in optical density compared with the only lactose samples.

### 2.6. Galectin-3C agglutinate Moritella viscosa

**Figure 7:**
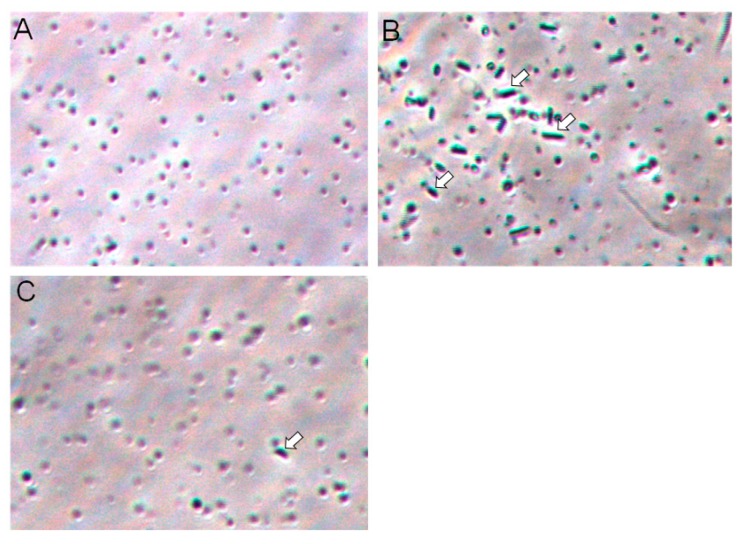
Bacterial agglutination. *Moritella viscosa* was incubated for 40 h in the absence (**A**) or presence of galectin-3C, 13 μg/mL (**B**). Rod shaped aggregates of *M. viscosa* (white arrows) were observed. In (**C**) bacteria was incubated in the presence of both galectin-3C and 0.5 M lactose, very few aggregates were observed.

Increased optical density can be the result of agglutination of bacteria. Forty-hour samples were microscopically compared, and bacterial agglutination was observed in the galectin-3C sample, but not in control and in very limited extent in galectin-3C with lactose (Figure 7). This suggest that agglutination in samples with high cell density increase the optical density.

### 2.7. Galectin-3C Modulate the Proteome of Moritella viscosa 

**Figure 8:**
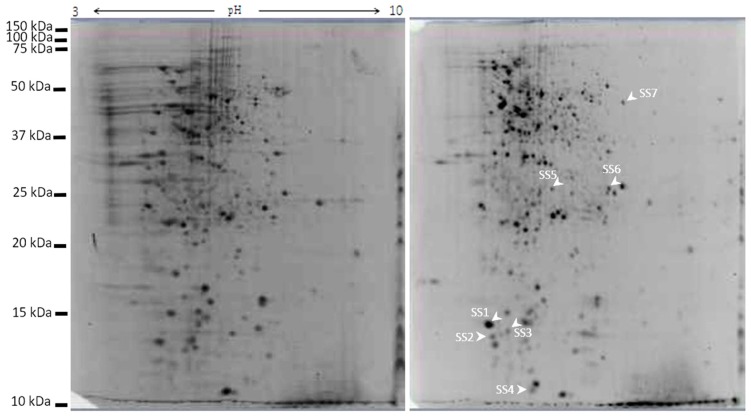
Two dimensional gels of *Moritella viscosa*. *Moritella viscosa* was incubated for 40 h in the presence (**A**) or absence of galectin-3C, 13 µg/mL (**B**), proteins were extracted and 100 µg was run on IPG-strips and then on a 15% SDS polyacrylamide gel.

**Figure 9:**
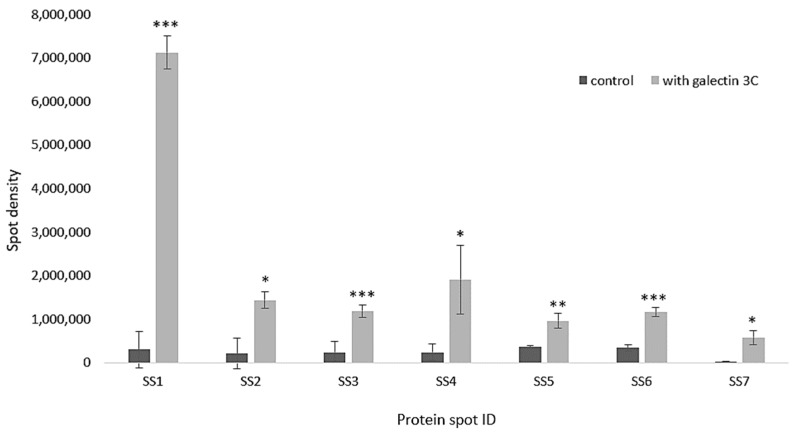
Differentially expressed bacterial proteins shown as spot intensities from two dimensional gels (*Y*-axis) in bar graphs based on the PDQuest Advanced 2D Analysis software. Error bars show standard deviation, *n* = 4, * means *p* < 0.05, ** means *p* < 0.01, and *** means *p* < 0.001.

*Moritella viscosa* incubated in the presence of galectin-3C had a modulated proteome compared to control samples without galectin-3C present. Gels were normalized and analyzed using PDQuest Advance 2D analysis software (BioRad). Seven spots were identified showing significant increased intensity (*p* < 0.05 and fold change more than 2) in two-dimensional gel electrophoresis gels (Figure 8 and Figure 9) of galectin-3C samples compared to control.

The spots were analyzed by MS-MS followed by Mascot homology search and identified as 50S ribosomal protein L7/L12 (two spots), 30S ribosomal protein S13, high-molecular weight cobalt-containing nitrile hydratase subunit alpha, multidrug transporter, 30S ribosomal protein S2, transcription termination factor Rho (Table 1). Changes in spots densities mean increased amounts of the protein in the spot. The spot that is increasing could be a post-translationally modified version of the protein, hence an increased spot density means that a change is made in protein modification(s) and/or total protein amount.

## 3. Discussion

Mucosal surfaces of animals including humans have living cells that are vulnerable for invasion of pathogens. The molecular defense system in fish skin include the mucus that both provides a physical barrier and is a reservoir for defense molecules [1,2]. One group of defense molecules is the lectins, glycan binding proteins with a large range of specificities and immune related functions. Galectins can for example both stimulate and inhibit inflammation and galectin-1, -2, -3, -4, -7, -8, -9, and -12 can all induce T-cell apoptosis [18]. Galectins can also work as opsonins to stimulate phagocytosis, they are produced and secreted by many cells including immune cell (reviewed in [19]).

The galectins that were previously characterized in skin and/or mucus are prototype galectins consisting of only the globular carbohydrate recognition domain [13,14,15], the size of our isolated protein (Figure 1) fits with the expected molecular weight of carbohydrate recognition domains of approximately 15 kD. However, the Atlantic salmon skin mucus galectin was identified as galectin-3 by mass spectrometry. Galectin-3 is a chimeric galectin with both a N-terminal domain and a C-terminal carbohydrate recognition domain, and an expected molecular weight of ~30 kD. The observed molecular weight of the isolated protein was however around 15 kD, as expected for prototype galectins. This discrepancy in observed and expected molecular weight was explained when further analysis showed that we isolated a truncated form, galectin-3C, with only the C-terminal carbohydrate recognition domain. In accordance with our observation, in humans galectin-3 (~30 kD) can also be found in a truncated form due to cleavage by metalloproteases [20], resulting in a 22 kD protein. Galectin-3 is a multifunctional protein, the protein’s location and activity is thought to be modulated by post-translational modifications and different interaction partners. In mammals including humans, results suggest that extracellular full length galectin-3 promotes transformation and metastasis of tumor cells, whilst galectin-3C can inhibit metastasis [21], showing that the post-translational cleavage of galectin-3 affects its function.

Galectin-3 does not have a signal peptide for translation on the endoplasmic reticulum, for later export out of cells through the endoplasmic reticulum-Golgi secretion pathway. It is thought to be exported from the cytosol by non-classical mechanism(s) [22] likely through exosomes [17]. The N-terminal of the protein is needed for secretion [17,23,24] and a sequence needed for import into multivesicular endosomes and export in exosomes, P(S/T)AP [17], is present once as PTAP in the N-terminal of salmon galectin-3. For humans it has been shown that the N-terminal domain is sensitive to metalloproteases [20]. Metalloproteases are present in the skin mucus of several fish species [25] including Atlantic salmon [26], and could hence be responsible for removal of the N-terminal resulting in truncated galectin-3 in skin mucus.

We found the highest expression of galectin-3 mRNA in skin and gills. High skin mRNA levels could suggest that that galectin-3C is made in its full-length form in skin cells, is exported with the N-terminal intact and then cleaved by metalloproteases in skin mucus. However, further studies are needed to confirm this hypothesis.

Galectin-3 can polymerize using its N-terminal usually to make pentamers [27]. Dimers can also be formed with interactions between two C-terminals [28]. The dimerization of the C-terminal part can be inhibited by the addition of glucan ligand [28]. Galectin-3C isolated in this study gives hemagglutination and bacterial agglutination in a lactose inhibitable manner.

*Moritella viscosa* is a bacterium that causes ulcers in the skin of Atlantic salmon [16]. The binding of galectin-3C to *Moritella viscosa* could be part of defense against the bacterium. Bacterial aggregates will have difficulties in penetrating host cells and can be sloughed off the fish with skin mucus during swimming. If the level of molecules that cause bacterial agglutination, including galectin-3C can be modulated for example with functional feeds, this holds a potential to reduce infection by pathogens that enter through mucosal surfaces. Agglutination of bacteria by immunoglobulin hinders bacterial colonization and also transfer of bacteria between animals [29]. Some galectins such as galectin-4 and 8 can directly kill bacteria [30], the killing activity is specific and restrained to only some bacteria [30]. Mucus galectin-3C did not inhibit growth of *Moritella viscosa* as assessed by measurement of turbidity nor did it produce clear zones in a zone of inhibition test [31] (results not shown). There was an increased optical density in the presence of galectin-3C at later time points (Figure 6), this could be explained by the formation of aggregates (Figure 7). This is in accordance with findings in a study of chitosan introduced aggregates of *Escherichia coli* [32]. Low concentrations of chitosan give abrupt increase in optical density in the *Escherichia coli* growth curve, but measurement of green fluorescence (from the bacterial strain’s green fluorescent protein (GFP)) amount indicates that there are fewer rather than more bacteria present than in the control samples. Scanning electron microscopy shows bacterial aggregates in samples with abrupt optical density increases, but not in control samples or at chitosan concentrations where no sudden increases in optical density was observed [32]. In the presence of lactose the bacteria grew slower, in accordance with the known inhibition of bacterial growth of carbohydrates due to hyperosmotic extracellular fluid and in accordance with a previous study showing no oxidative production of acid for the bacteria in the presence of lactose [33]. Lactose inhibited galactose-3C induced hemagglutination. Limited bacterial agglutination was observed in samples incubated with both galectin-3C and lactose. In both the presence and absence of lactose, galectin-3C aggregate formation formed at later time points. This could indicate that the aggregates are forming on the galectins as the bacteria divide, and not by simple aggregation of already present bacteria. Aggregate formation by bacterial division on immunoglobulin A has been shown to be responsible for aggregate formation in the mucosal surface in the gut [34].

To further study the interaction between galectin-3C and *Moritella viscosa*, the bacteria incubated with and without galectin-3C was studied by two-dimensional gel electrophoresis. Increased spot densities for multi drug transporter, high-molecular weight cobalt-containing nitrile hydratase subunit alpha, transcription termination factor Rho, and ribosomal proteins, were found. The multidrug transporter is used to export harmful substances from bacteria, and also is involved in cell-cell contact and biofilm formation [35]. Nitrile hydratases hydrates harmful nitriles to amides, hence protect the bacteria. It can be upregulated in stressed bacteria as shown in bacteria treated with the pesticide linuron [36]. Transcription termination factor Rho is as the name says involved in termination of transcription, it is targeted by the antibiotics bicyclomycin. Interestingly, knock down of Rho has been linked to increased *Staphylococcus aureus* virulence [37], indicating that bacteria under less favrable conditions increase their virulence after Rho silicing.

The bacterial ribosome consists of two parts, the 50S and the 30S subunits. Since ribosomes are key for cell growth and survival, targeting them can kill cells, there are antibiotics targeting both the 30S [38] and 50S [39] subunits. Galectin-3C induced spot differences in 50S ribosomal protein L7/L12 (two spots), 30S ribosomal protein S13, and 30S ribosomal protein S2. Typically changes in ribosomal subunits for example have been observed when bacteria are in non-favorable conditions and proteomics changes in ribosomal subunits have been found with antibiotics that are inhibitors of translation elongation [40].

Overall, the changes observed in *Moritella viscosa* indicate that galectin-3C is not simply binding to the bacteria and aggregating them, but are also inducing changes in signaling pathways to change proteins in the bacterial cytoplasm. Further studies are needed to understand the interaction between galectin-3C in the host immune system and pathogens, and whether galectin-3 and galectin-3C have the same function.

In mammals galectins are focused upon because they have important roles in human disease as well as regulating functions such as inflammation and metabolism [18]. For experimental purposes they are targeted with inhibitors and their amounts increased by injection. Galectins from other species, including fish, could have potential to be used to inhibit or kill bacteria and to promote wound healing. A galectin from Japanese eel *Anguilla japonica*, AJL-1 inhibits biofilm formation by human bacteria important for periodontal disease [41]. Fish skin has antibacterial and wound healing promoting properties and are appealing for use in human medicine as there is less risk of disease transfer including prions than when mammalian grafts are being used [42]. Since galectins can kill bacteria [30], inhibit bacterial biofilm formation [41] and have wound healing properties [43], at least some of the wound healing property of fish skin could be due to the presence of galectin. Galectins could be isolated from by-products from industrial processing of fish. However, additional research is needed to establish the effectivity and biosafety of fish galectins on human pathogens and human wound healing.

## 4. Materials and Methods 

### 4.1. Fish and Their Maintenance

Atlantic salmon, AquaGen Strain: DIPLOID R*E QTL IPN/PD + ILA/IPN/PD arrived from Cermaq Hopen; Norway as smolt, and were reared in indoor tanks at Mørkvedbukta research station, Nord University, Bodø, Norway. The fishes were maintained at temperature 12 °C in a sea water flow through system and fed to saturation on a commercial diet (Skretting, Norway).

### 4.2. Sampling of Skin Mucus and Tissues

Atlantic salmon weighing approximately 1 kg were used for sampling. Fishes were anesthetized with MS-222 (70 mg/L), mucus was scraped from the skin on the dorsal and ventral body surface of Atlantic salmon with a sterile glass slide avoiding the anal region to prevent fecal contamination. The skin mucus samples were stored in −80 °C freezer for later use. Tissue samples (approximately two mm^3^) of skin, muscle, gill, foregut, hindgut, stomach, liver, head kidney, and spleen were collected and immediately frozen in liquid nitrogen and moved to a −80 °C freezer for storage until further analysis. National guidelines for animal rearing and handling procedures from The Norwegian Food Safety Authority (www.mattilsynet.no/language/english/about_us/) were followed. For use of animals for sampling of organs and tissues only i.e., no experimenting on live animals, an ethical approval is not needed under local legislation.

### 4.3. Purification of Salmo salar Galectin-3 from Skin Mucus

#### 4.3.1. Preparation of Skin Mucus Sample for Purification

Skin mucus samples stored at −80 °C were thawed on ice. Five ml of mucus sample in a 50 mL tube was mixed with protease inhibitor (10 μL/mL) (GE Healthcare, Chicago, IL, USA). The sample was diluted by adding 3 volumes of binding buffer (20 mM Tris-HCl, 10 mM CaCl_2_, 10 mM MgCl_2_, 0.5 M NaCl, pH-7.5) and mixed well by pipetting. The sample was sonicated 3 times 5 s using an ultrasonic processor (SONICS Vibracell VCX750, Newtown, CT, USA), with cooling in an ice bath between sonications. The sonicated mucus sample was centrifuged at 14,000× *g* for 30 min at 4 °C to remove debris. The supernatant was used for purification.

#### 4.3.2. Binding of Skin Mucus Protein to α-Lactose Agarose

The purification was done by following the protocol described by Rajan et al. [13] with minor modifications. Approximately 5 mL of α-lactose agarose resin (Sigma-Aldrich, St. Louis, MO, USA) in a 50 mL tube was washed twice with 3 volumes of 15 MΩ/cm analytic grade water. Washed resin was equilibrated with 3 volumes of binding buffer at 4 °C, and 20 mL of prepared skin mucus supernatant was added and allowed to bind for 2 h at 4 °C under rotation, followed by three times washing with binding buffer. The resin was loaded to a 10 ml Bio-Rad gravity purification column. The lactose binding protein was eluted manually using elution buffer (20 mM Tris-HCl, 0.5 M NaCl, 0.5 M α-lactose).

Ten fractions of eluate, 500 µL each, were collected and the protein concentration in each fraction quantified by Qubit® Protein Assay Kit in a Qubit® fluorometer (Life Technologies, Camarillo, CA, USA). Each fraction containing protein was then desalted.

#### 4.3.3. Desalting of Purified Protein Using Sephadex G-15

Dry resin of Sephadex G-15 (GE Healthcare, Life Sciences, Buckinghamshire, UK) were washed and let to swell overnight at 4 °C in analytic grade water (15 MΩ/cm). Two ml of swollen resin in a 10 mL Bio-Rad gravity purification column was equilibrated with PBS (phosphate buffered saline). Five hundred µl of eluted fraction from α-lactose agarose column was loaded to the equilibrated Sephadex G-15 column. Ten fractions (0.5 mL each) were eluted from the column. Eluted fractions containing protein were pooled and up concentrated using 3K cut off centrifugal filters (VWR, Radnor, PA, USA). The protein after concentration was quantified using Qubit^®^ Protein Assay Kit and Qubit fluorometer (Life Technologies, Camarillo, CA, USA) and kept in −20° C for further use.

### 4.4. SDS-PAGE

The protein profile of the purified product was determined on a 15% poly acrylamide gel [44]. In short sample was mixed with protein loading buffer (Laemmli sample buffer [44] mixed with β-mercaptoethanol, Bio-Rad, Irvine, CA, USA) and incubated at 95 °C for 5 min before loading to the gel. The gel was run in Bio-Rad Mini PROTEAN® Tetra cell, at 100 V. Precision Plus, Kaleidoscope^TM^ (Protein^TM^ Standards, Bio-Rad) protein marker was used as a molecular weight standard. Gels were stained in colloidal Coomassie G-250 (0.08% Coomassie Blue G-250 in buffered 20% methanol) overnight, destained using analytic grade water, and digitalized (ChemiDoc^TM^ XRS System, Bio-Rad).

### 4.5. LC/MS-MS Analysis

Protein band (Figure 1) in the gel was excised, reduced, alkylated and trypsinized to obtain a peptide mixture as described elsewhere [45]. LC-MS/MS analysis of the peptide mixture was done with ESI-Quad-TOF, USA and Q-Exactive, Thermo Scientific. Peak lists (pkl) were obtained by Protein Lynx Global server software (version 2.1, Waters Corporation, Milford, MA, USA).

### 4.6. Bioinformatic Analysis of Mass Spectrometry Files

The peak list files obtained were analyzed using MASCOT MS/MS Ions search (http://www.matrixscience.com) against NCBIprot 20,180,429 (152462470 sequences; 55858910152 residues) or if no hits where obtained then SwissProt 2019_09 (561176 sequences; 201,758,313 residues). Lactose-binding isolated protein pkl-files were searched against Actinopterygii (ray-finned fishes) (2346792 sequences) and *Moritella viscosa* pkl-files against Bacteria (Eubacteria) (114425735 sequences). The Mascot parameters set were fixed carbamidomethyl (C) modification, variable oxidation (M) modification, peptide mass tolerance of 100 ppm, fragment mass tolerance of 0.1 Da, monoisotopic mass values, maximum 1 missed cleavages by the enzyme trypsin.

### 4.7. Hemagglutination and Inhibition Assays

Hemagglutination activity was measured by using horse erythrocytes from Statens Serum Institute, Copenhagen, Denmark. The horse blood was treated with 0.05% trypsin EDTA, and fixed in 1% glutaraldehyde as described by Nowak et al. [46]. Serial two-fold dilutions of the purified protein (200 µg/mL) was used. The assay was performed in a U-bottomed microtiter plate (VWR, Norway). Each well contained 25 µL of 1% BSA in PBS, 25 µL of PBS, 25 µL of undiluted or diluted protein sample and 25 μL of 4% erythrocyte suspension. In the negative control the protein sample was replaced by 25 µL of PBS and for hemagglutination inhibition assay PBS was replaced with 0.5 M lactose in PBS. Samples were mixed for 30 s at slow speed in a microplate vortex (Genie^®^, Scientific industries, INC, Long islands, Bohemia, NY, USA). The plate was incubated for an hour at room temperature and hemagglutination activity was visually observed and documented by photography.

### 4.8. Tissue Distribution of Galectin-3 Gene Expression

#### 4.8.1. RNA Extraction and cDNA Synthesis

RNA from gill, skin, muscle, stomach, foregut, hindgut, spleen, head kidney, and liver of healthy Atlantic salmon were extracted from frozen (liquid nitrogen and −80 °C) tissue using EZNA total RNA kit (Omega Bio-Tek, Norcross, GA, USA) following the manufacturer’s manual. Extracted RNA was quantified with Qubit^®^ RNA BR Assay kit and Qubit^®^ fluorometer (Life technologies, Carlsbad, CA, USA). The total RNA integrity was assessed by running a 1.2% agarose gel. cDNA was synthesized using QuantiTect Reverse Transcription kit with integrated removal of genomic DNA contamination (Qiagen, Germantown, MD, USA) following the manufacturer’s protocol.

#### 4.8.2. Primer Design

The mRNA sequence of Atlantic salmon (*Salmo salar*) galectin-3, (Accession No: NM_001140833 GI: 213514683) was retrieved from NCBI (http://www.ncbi.nlm.nih.gov). The sequence was blasted against NCBI Primer blast (http://www.ncbi.nlm.nih.gov/tools/primer-blast/) to pick primers specific for galectin-3 (gene symbol: *leg3*). The Primers obtained were analyzed for melting temperature and secondary structures (hairpins, self-dimers, cross dimers, palindromes, repeats and runs) using the Net Primer http://www.premierbiosoft.com/netprimer) software. Two sets of primers having minimum secondary structures and suitable thermal conditions were chosen for the study (Table 2). 

#### 4.8.3. Reverse Transcription-qPCR for Tissue Distribution Analysis

Reverse transcription-qPCR using the primers in Table 2 was performed to assess the distribution of *leg3* expression in tissues of Atlantic salmon.

The thermocycling condition used was: holding stage of 20 s at 95 °C for activation of Taq DNA polymerase, followed by 45 cycles of denaturation at 95 °C for 3 s, annealing at 60 °C for 30 s, a data acquisition step of 15 s at 60 °C during the annealing stage in each cycle. The qPCR reaction mixtures in 96 wells plate (Applied Biosystems, Foster City, CA, USA) wells were 10 µL containing 5 µL of SYBR Green master mix (Applied Biosystems, USA), 4 µL of 25x diluted cDNA, 0.5 µL of each primer. The prepared 96 well plates were covered with an optical adhesive (Applied Biosystems, Foster City, CA, USA), centrifuged for a minute before analysis using StepOneplus real time PCR system (Applied Biosystems, Foster City, CA, USA). Each reaction was run in duplicates with appropriate standards (a fivefold dilution series of pooled cDNA) and negative control (no template reaction). The same reaction set up and PCR conditions were used for the reference gene *elongation factor 1 alfa* [47,48].

### 4.9. Effect of *Salmo salar* Galectin-3C on the Proteome of *Moritella Viscosa* and Agglutination of the Bacteria

#### 4.9.1. Bacterial Strain and Experimental Set Up

Stock culture of *M. viscosa* (Glomfjord isolate, in house isolate from an outbreak of winter ulcers in Glomfjord, Norway) stored at −80 °C was used in the study. A loop of bacteria from stock was streaked on a nutrient agar plate supplemented with 3% TSB and 1.5% NaCl and incubated at 15 °C. A colony from the plate was picked and inoculated in media containing 3% TSB (Tryptic soy broth, Fluka, Sigma-Aldrich, St. Louis, MO, USA) supplemented with 1.5% NaCl at 15 °C.

The experimental media included 3% TSB, 1.5% NaCl supplemented with 13 μg of purified galectin-3C per ml of media. To observe the inhibitory effect of lactose in one set up 0.5 M lactose was added to the media along with galectin-3C (13 μg/mL of medium). One more set up was made by adding only 0.5 M lactose but no galectin-3C. In control (3% TSB + 1.5% NaCl) samples PBS was added in place of galectin-3C to make the volume equal in all set ups. A bacterial culture of 0.6 OD was used as inoculum for the experiment. The experiment was done in duplicates at 15 °C. Cell growth of bacteria was measured at 590 nm at interval of every 4 h. Samples for two-dimensional gel electrophoresis (2D) analysis were taken at 40 h. For 2D the samples were centrifugated (4,000× *g*, 10 min, 4 °C), the pellets washed two times in a low salt buffer (3 mM KCl, 1.5 mM KH_2_PO_4_, 20 mM NaCl and 9 mM NaH_2_PO_4_) [49], and stored at −80 °C.

#### 4.9.2. Sample Preparation for 2D Analysis

Samples for 2D was further prepared following a published protocol [49] with small modifications. Briefly, bacterial cell pellets was thawed in lysis buffer (9 M urea, 4% CHAPS, 1% DTT and 0.8% Bio-Lyte 3/10 ampholyte (Bio-Rad, Irvine, CA, USA)), sonicated (2 × 5 s) using an ultrasonic processor (SONICS Vibracell VCX750, Newtown, CT, USA). One microliter of benzonase nuclease (Benzonase^TM^ Nuclease, Sigma-Aldrich, St. Louis, MO, USA) was added to degrade DNA and reduce the viscosity of the sample. The sample was dialyzed using 3 kD cut off Nanosep spin columns (VWR, Oslo, Norway) following the manufacturer’s protocol. Protein concentration was measured using Qubit^®^ Protein Assay Kit and Qubit^TM^ fluorometer (Life Technologies, Carlsbad, CA, USA).

A volume of sample giving 100 µg protein was mixed with rehydration buffer (7 M urea, 2 M thiourea, 4% CHAPS, 15 mM DTT, 0.5% Biolyte 3-10, 10% glycerol and 0.001% bromophenol blue) to a total volume of 300 µL. This volume was used to rehydrate 17 cm IPG strip (pH 3-10, Bio-Rad, Irvine, CA, USA) overnight following the manufacturer’s protocol.

Rehydrated IPG strips were isoelectricaly focused in Protean IEF cell (Bio-Rad, USA) to total 60,000 volt hours at a maximum of 10,000 V using three steps of slow ramping at a constant temperature of 20 °C [50]. Next, the electro focused IPG strips were sequentially reduced for 15 min and alkylated for 15 min in equilibration buffer (6 M urea, 0.375 M Tris- HCl (pH 8.8), 2% SDS, 20% glycerol) containing 2% *w/v* DDT or 3% *w/v* iodoacetamide respectively. The equilibrated strips were subjected to a second dimension run in 12.5% polyacrylamide gels using Bio-Rad Protean IIxii system (Irvine, CA, USA). After completion of the run gels were stained (Sypro^®^ Ruby Protein gel stain, Life technologies, Carlsbad, CA, USA) and images documented by the ChemiDoc^TM^ XRS system (Bio-Rad). The documented gels were analyzed with PDQuest^TM^ Advanced 2D analysis software (Bio-Rad), including only spots with a fold change more than 2 and *p* < 0.05. The PDQuest software uses a local regression model to normalize the spots. The normalization compensates the gel to gel variation in spots that are not related to protein variation such as inconsistency in staining, and differences in sample density. To test the significance of the spots the default *t*-test from PDQuest was used.

Differentially expressed spots were manually excised on a blue light transilluminator (Safe Imager™ 2.0 Blue-Light Transilluminator, Life technologies, USA) and subjected for LC-MS/MS analysis. The LC-MS/MS and MASCOT analyses for protein identification were performed as described in Section 4.5 and Section 4.6.

#### 4.9.3. Bacterial Agglutination

*Moritella viscosa* was grown in media supplemented with purified galectin-3C or with PBS (control) as described in 4.9.1. To check the inhibition of bacterial agglutination, 0.5 M of lactose in addition to galectin-3C was present during the incubation (as described in 4.9.1). Forty hours bacterial cultures (OD < 1) were smeared on sterile glass slides and observed under light microscope (Leica microsystems).

## Figures and Tables

**Table 1:** List of proteins that are differentially expressed in *Moritella viscosa* between samples incubated for 40 h without (control) and with galectin-3C.

Spot ID	Accession ID/Database	Protein Name/Organism/ Major Protein Domain	Calculated pI/Nominal Mass (Mr)	Mascot ion Score/ *p*-Value	SC (%)/ UP Score	ST	Peptide Sequences
SS1	WP_002958882 NCBIprot	50S ribosomal protein L7/L12 *Pseudoalteromonas* Ribosomal_L7_L12 superfamily	4.45/12194	407/ *p* < 0.01	50/406	69	KDLTEAGAEVEVK KEGVSKEEAEALAKD KTEFDVILTGAGANKV KFGVTAAAAMVAGPA-AEAAEEKT
SS2	Q3ILQ0 NCBIprot	50S ribosomal protein L7/L12/*Pseudoalteromonas haloplanktis* Ribosomal_L19 superfamily	4.45/12222	161/ *p* < 0.008	19/161	71	KALVEAAPTPVKE KDLTEAGAEVEVK
SS3	Q3IJK6 Swissprot	30S ribosomal protein S13/*Pseudoalteromonas haloplanktis* Ribosomal_S13 superfamily	10.95/13162	123/ *p* < 0.0005	11/123	72	KIGELSDETLDVLRD
SS4	P21219 SwissProt	High-molecular weight cobalt-containing nitrile hydratase subunit alpha/*Rhodococcus rhodochrous*	4.85/22991	58/ *p* < 0.05	6/58	37	RDFGFDIPDEVEVRV
SS5	WP_016707503 NCBIpro	multidrug transporter/*Pseudoalteromonas* SapC superfamily	5.38/26438	178/ *p* < 0.01	15/178	70	KEALVSFIEFSHVTEAFTKY KYLADKELLVAQTLTVDIKG
SS6	WP_045111824 NCBIprot	30S ribosomal protein S2/*Moritella viscosa* RPS2 superfamily	6.60/26834	169/ *p* < 0.01	12/169	66	KLYAGAVAASVNEGRN RNQDIAAQAESDFIEEAK
SS7	CCQ10658 NCBIprot	Transcription termination factor Rho/*Pseudoalteromonas luteoviolacea* Rho superfamily	6.55/47155	149/ *p* < 0.01	6/77	66	KGEVIASTFDEPASRH KILFENLTPIHANERL

**Table 2:** Galectin-3 primers used in the study.

Gene Name	Gene Symbol	Sequence (5′>3′)
Galectin-3	*leg3*	Forward: GAGTTCAAACACCGCATCCG Reverse: GGCTGAAACCAACCCTGCTA

## Supplementary Materials

The following are available online at https://www.mdpi.com/1660-3397/18/2/102/s1, Supplementary file showing peptides identified in galectin-3C by mass spectrometry.
