# Supplementary material for: A Truncated Galectin-3 Isolated from Skin Mucus of Atlantic Salmon Salmo salar Binds to and Modulates the Proteome of the Gram-Negative Bacteria Moritella viscosa
Source: Mar Drugs. 2020 Feb 4;18(2):102. doi: 10.3390/md18020102 (PMC7074318; doi:10.3390/md18020102)
Supplement: Supplementary file 1 [file marinedrugs-18-00102-s001.pdf]

Patel et al “A truncated galectin-3 isolated from skin mucus of Atlantic salmon *Salmo salar* binds to and modulates the proteome of the Gram negative bacteria *Moritella viscosa*” SUPPLEMENTARY FILE

Full stops indicate trypsin cleavage sites. Trypsin cleave on the carboxyl side of the amino acids lysine or arginine. Peptides identified by the mass spectrometers are the amino acids between the full stops.

Peptides identified in galectin 3 with ESI-Q-TOF and mascot search.

K.MFTINLTK.G

R.NSQIGNTWGK.E

K.IMCTNSEFK.V

K.GNDIAMHVNPR.F

Peptides identified in galectin 3 with Q- Exactive and mascot search

K.HLGIYNDVTLTSVEIDKL (C-terminal of protein)

K.MPYDLNLPNGCYDK.M

K.VAVNSSHILEFK.H

K.IMCTNSEFK.V

K.TIVRNSQIGNTWGK.E

R.NSQIGNTWGKEER.E

R.NSQIGNTWGK.E

K.MFTINLTK.G

K.GNDIAMHVNPR.F

R.EHNHFPFIQGQPFEMK.I
